# 

**Published:** 1847-04

**Authors:** 


### THE BRITISH AND FOREIGN MEDICAL REVIEW,

OR

### Quarterly Journal

OF

### PRACTICAL MEDICINE AND SURGERY.

---

EDITED BY

JOHN FORBES, M.D. F.R.S. F.G.S.

LONDON:

